# Supplementary material for: The ALS-Associated FUS (P525L) Variant Does Not Directly Interfere with Microtubule-Dependent Kinesin-1 Motility
Source: Int J Mol Sci. 2021 Feb 28;22(5):2422. doi: 10.3390/ijms22052422 (PMC7957795; doi:10.3390/ijms22052422)
Supplement: Supplementary file 1 [file ijms-22-02422-s001.pdf]

International Journal of
Molecular Sciences

Supplementary Materials

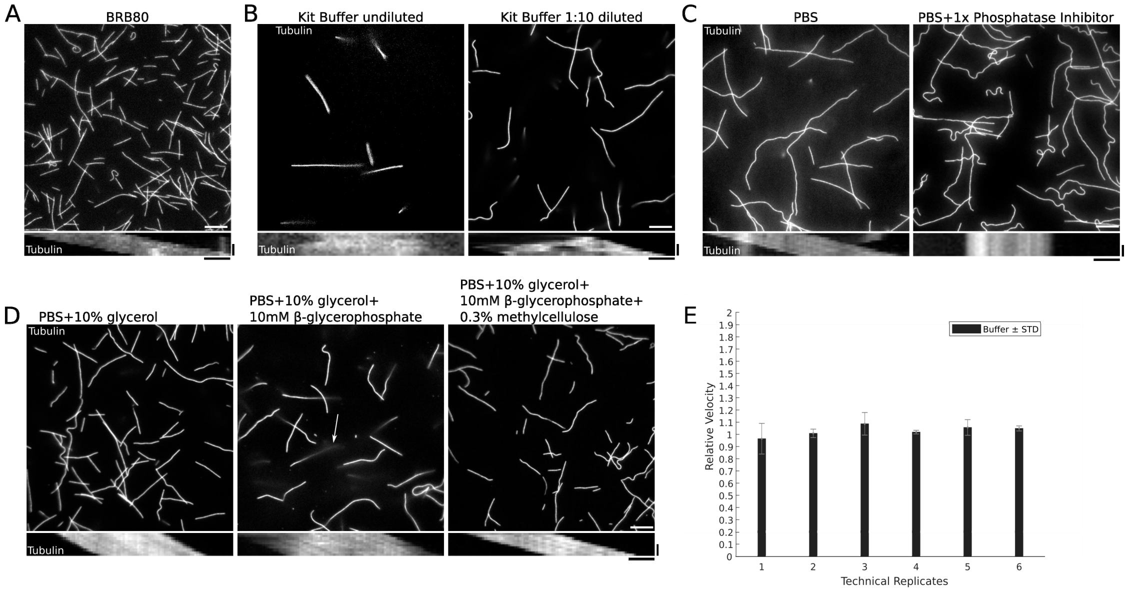

Figure S1. Optimal buffer conditions for protein-enriched, kinesin-1-dependent microtubule gliding motility assays consist of PBS supplemented with 10% glycerol, 10 mM β-glycerophosphate, and 0.3% methylcellulose. Top of each panel: Kinesin-1-dependent gliding of rhodamine-labeled microtubules: (**A**) in presence of BRB80 alone, (**B**) at indicated dilutions of whole cell lysis buffer utilized in a Nuclear Extract Kit for whole cell lysates (Active Motif, Waterloo, Belgium), (**C**) in presence of PBS alone or supplemented with 1x phosphatase inhibitor cocktail (Halt^{tM}, Thermo Fisher Scientific, Waltham, MA, USA), (**D**) in presence of PBS supplemented with 10 % glycerol, additional 10 mM β-glycerophosphate (white arrow marks a detaching microtubule), and in combination with 0.3% methylcellulose. Scale bar = 10 µm. Bottom of each panel: Representative kymographs showing the kinesin-1-dependent movement of microtubules in the respective conditions. Horizontal scale bar = 2 µm, vertical = 5 s. (**E**) Relative kinesin-1-dependent microtubule gliding velocities (see Materials and Methods) of six technical replicates in standard motility buffer.

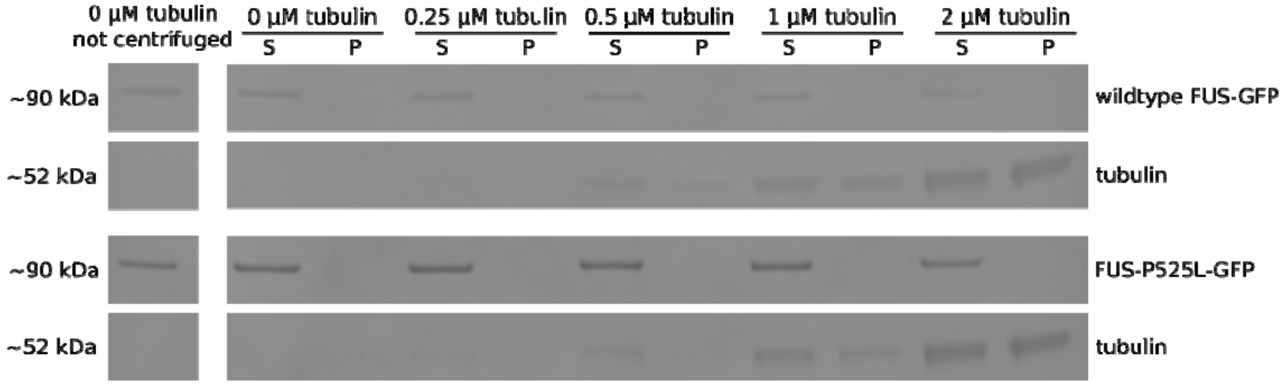

Figure S2. Recombinant wildtype FUS-GFP and FUS-P525L-GFP does not bind to microtubules. Pull-down assay of microtubules at indicated concentrations of tubulin incubated with 500 nM of either recombinant wildtype FUS-GFP or FUS-P525L-GFP.

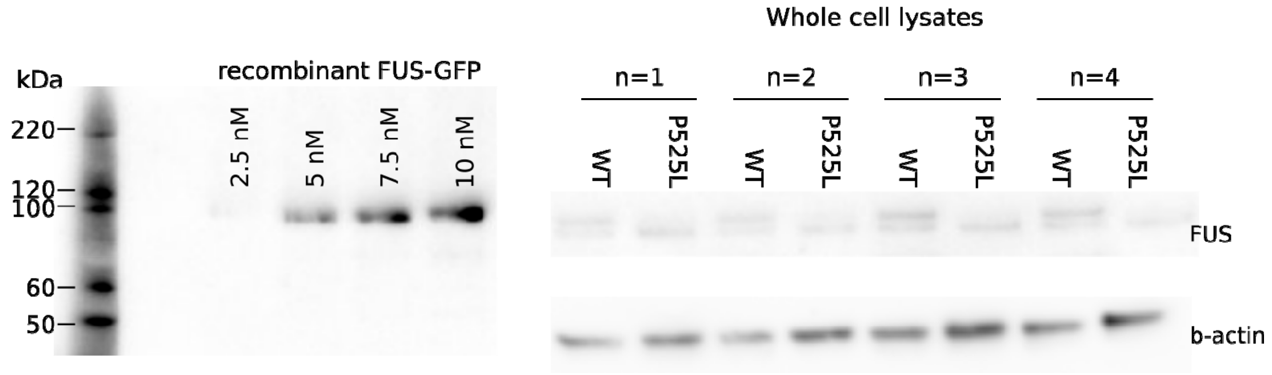

Figure S3. Western blot of FUS-GFP variants expressed in whole cell lysates of spinal motor **neurons.** Representative western blot of recombinant wildtype FUS-GFP at indicated concentrations and four biological replicates (n) of whole cell lysates obtained from spinal motor neurons expressing either wildtype FUS-GFP or FUS P525L GFP.

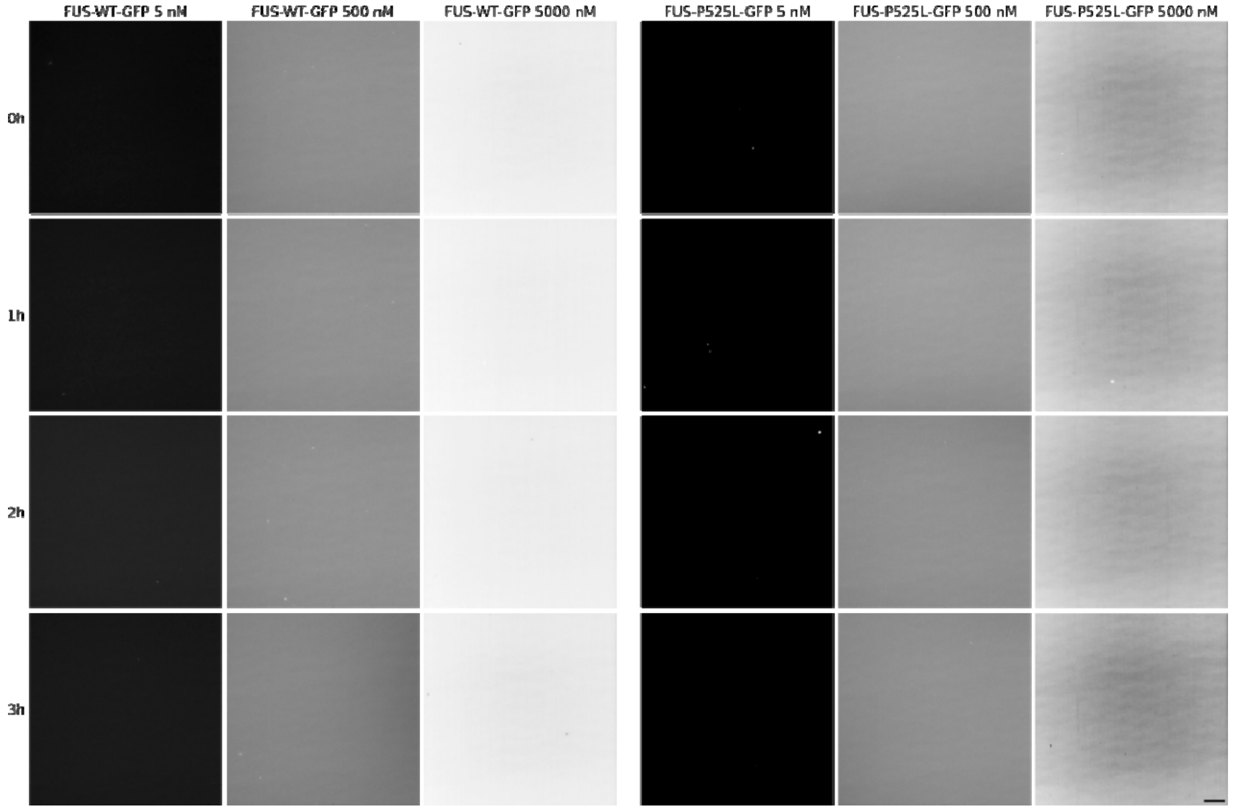

Figure S4. Recombinant wildtype FUS-GFP and FUS-P525L-GFP do not aggregate over the course of three hours in our assay conditions. Free recombinant wildtype FUS-GFP (left three columns) and ALS-associated FUS-P525L-GFP (right three columns) in standard motility buffer at indicated concentrations (left to right) and time points (top to bottom). Scale bar = 10 µm.

Table S1. Significance values (*p*-values) for kinesin-1-dependent microtubule gliding in the presence of recombinant human wildtype FUS-GFP or FUS-P525L-GFP, standard motility buffer or protein control (BSA) over the course of three hours at indicated concentrations and time points (referring to Figures 3).

**5 nM**	**FUS-WT-GFP**	**FUS-P525L-GFP**	**BSA**	**Time**
Buffer	0.9991	0.9885	0.9502	
FUS-WT-GFP	-	0.9977	0.9079	0 h
FUS-P525L-GFP	-	-	0.8285	
Buffer	>0.9999	>0.9999	>0.9999	
FUS-WT-GFP	-	>0.9999	>0.9999	1 h
FUS-P525L-GFP	-	-	>0.9999	
Buffer	>0.9999	0.9985	0.9976	
FUS-WT-GFP	-	0.9131	0.9024	2 h
FUS-P525L-GFP	-	-	>0.9999	
Buffer	>0.9999	0.9975	0.9933	
FUS-WT-GFP	-	0.9968	0.9928	3 h
FUS-P525L-GFP	-	-	>0.9999	
**500 nM**	**FUS-WT-GFP**	**FUS-P525L-GFP**	**BSA**	**Time**
Buffer	0.3289	0.0135	>0.9999	
FUS-WT-GFP	-	0.4325	0.2927	0 h
FUS-P525L-GFP	-	-	0.0111	
Buffer	0.1702	0.0543	>0.9999	
FUS-WT-GFP	-	>0.9999	>0.9999	1 h
FUS-P525L-GFP	-	-	0.5244	
Buffer	0.2112	0.1338	0.8306	
FUS-WT-GFP	-	0.9952	0.7023	2 h
FUS-P525L-GFP	-	-	0.5587	
Buffer	0.1695	0.3686	0.871	
FUS-WT-GFP	-	0.9684	0.469	3 h
FUS-P525L-GFP	-	-	0.7612	
**5000 nM**	**FUS-WT-GFP**	**FUS-P525L-GFP**	**BSA**	**Time**
Buffer	0.1765	0.5127	0.9899	
FUS-WT-GFP	-	0.8327	0.0971	0 h
FUS-P525L-GFP	-	-	0.3302	
Buffer	0.0162	>0.9999	>0.9999	
FUS-WT-GFP	-	0.3011	0.016	1 h
FUS-P525L-GFP	-	-	0.4378	
Buffer	0.0465	0.3882	0.9997	
FUS-WT-GFP	-	0.6555	0.0375	2 h
FUS-P525L-GFP	-	-	0.3377	
Buffer	0.0449	0.3301	0.9964	
FUS-WT-GFP	-	0.6869	0.0691	3 h
FUS-P525L-GFP	-	-	0.4408	

Table S2. Significance values (*p*-values) for kinesin-1-dependent microtubule gliding in the presence of recombinant human 2N4R tau-GFP at indicated concentrations (referring to Figure 2).

Tau-GFP	1 nM	5 nM	10 nM	15 nM	25 nM	50 nM	75 nM	100 nM
0 nM	>0.9999	>0.9999	>0.9999	0.8786	0.016	0.0005	0.0004	0.0004
1 nM	-	>0.9999	>0.9999	0.909	0.0189	0.0005	0.0005	0.0005
5 nM	-	-	>0.9999	0.9537	0.0258	0.0007	0.0007	0.0007
10 nM	-	-	-	0.9828	0.0366	0.001	0.001	0.001
15 nM	-	-	-	0.9828	0.2066	0.0067	0.0063	0.0062
25 nM	-	-	-	-	-	0.6482	0.6255	0.6216
50 nM	-	-	-	-	-	-	>0.9999	>0.9999
75 nM	-	-	-	-	-	-	-	>0.9999

Table S3. Significance values (*p*-values) for kinesin-1-dependent microtubule gliding in the presence of neural cell lysates of wildtype FUS-GFP or FUS-P525L-GFP cell lines, standard motility buffer or protein control (BSA) at indicated concentrations (referring to Figure 4B).

	FUS-WT-GFP	FUS-P525L-GFP	BSA	Concentration
Buffer	0.0691	0.0544	0.3116	
FUS-WT-GFP	-	0.9992	0.8558	50 ng/µL
FUS-P525L-GFP	-	-	0.7956	
Buffer	0.9973	0.3495	0.48	
FUS-WT-GFP	-	0.2831	0.4451	80 ng/µL
FUS-P525L-GFP	-	-	0.998	
Buffer	0.827	0.5112	0.1648	
FUS-WT-GFP	-	0.9117	0.4083	110 ng/µL
FUS-P525L-GFP	-	-	0.7726	
Buffer	0.9889	0.5873	0.5245	
FUS-WT-GFP	-	0.6499	0.5884	140 ng/µL
FUS-P525L-GFP	-	-	0.9891	

Table S4. Significance values (*p*-values) for kinesin-1-dependent microtubule gliding in the presence neural cell lysates of wildtype FUS-GFP or FUS-P525L-GFP cell lines, standard motility buffer or protein control (BSA) at a concentration of 80 ng/µL supplemented with 2N4R tau-GFP and the indicated concentrations (referring to Figure 4C).

	Tau-GFP	FUS-WT-GFP	FUS-P525L-GFP	BSA	Concentration of tau-GFP
Buffer	>0.9999	0.9876	0.9974	>0.9999	
Tau-GFP	-	0.9544	0.9895	>0.9999	0 nM
FUS-WT-GFP	-	-	0.9993	0.9279	
FUS-P525L-GFP	-	-	-	0.9786	
Buffer	>0.9999	0.967	0.9748	>0.9999	
Tau-GFP	-	0.9223	0.9416	0.9991	1 nM
FUS-WT-GFP	-	-	>0.9999	0.8187	
FUS-P525L-GFP	-	-	-	0.8505	
Buffer	0.9997	0.8977	0.9003	>0.9999	
Tau-GFP	-	0.858	0.8625	0.9989	5 nM
FUS-WT-GFP	-	-	>0.9999	0.719	
FUS-P525L-GFP	-	-	-	0.725	
Buffer	0.997	0.2203	0.3247	0.9987	
Tau-GFP	-	0.0966	0.1882	>0.9999	10 mM
FUS-WT-GFP	-	-	0.9978	0.0775	
FUS-P525L-GFP	-	-	-	0.1555	
Buffer	0.7365	0.1383	0.0462	0.977	15 nM
Tau-GFP	-	0.4992	0.1674	0.8932	

FUS-WT-GFP	-	-	0.9636	0.0992	
FUS-P525L-GFP	-	-	-	0.0182	
Buffer	0.0002	<0.0001	0.0029	0.0002	25 nM
Tau-GFP	-	0.9991	0.7958	>0.9999	
FUS-WT-GFP	-	-	0.6478	0.9995	
FUS-P525L-GFP	-	-	-	0.776	
Buffer	<0.0001	<0.0001	<0.0001	<0.0001	50 nM
Tau-GFP	-	>0.9999	>0.9999	>0.9999	
FUS-WT-GFP	-	-	>0.9999	>0.9999	
FUS-P525L-GFP	-	-	-	>0.9999	
Buffer	<0.0001	<0.0001	<0.0001	<0.0001	75 nM
Tau-GFP	-	>0.9999	>0.9999	>0.9999	
FUS-WT-GFP	-	-	>0.9999	>0.9999	
FUS-P525L-GFP	-	-	-	>0.9999	
Buffer	<0.0001	<0.0001	<0.0001	<0.0001	100 nM
Tau-GFP	-	>0.9999	>0.9999	>0.9999	
FUS-WT-GFP	-	-	>0.9999	>0.9999	
FUS-P525L-GFP	-	-	-	>0.9999	
